# Prediction and detection of human epileptic seizures based on SIFT-MS chemometric data

**DOI:** 10.1038/s41598-020-75478-8

**Published:** 2020-10-27

**Authors:** Amélie Catala, Cecile Levasseur-Garcia, Marielle Pagès, Jean-Luc Schaff, Ugo Till, Leticia Vitola Pasetto, Martine Hausberger, Hugo Cousillas, Frederic Violleau, Marine Grandgeorge

**Affiliations:** 1 Association Handi’Chiens, 13 Rue de l’Abbé Groult, Paris, France; 2 Univ Rennes, Normandie Univ, CNRS, EthoS (Éthologie animale et humaine), UMR 6552, 35000 Rennes, France; 3 Laboratoire de Chimie Agro-industrielle (LCA), Université de Toulouse, INRA, University of Toulouse, National Polytechnic Institute of Toulouse, Ecole d’ingénieurs de Purpan, Toulouse, France; 4 Equipe Physiologie, Pathologie et Génétique Végétales (PPGV), University of Toulouse, National Polytechnic Institute of Toulouse, Ecole d’ingénieurs de Purpan, 75 voie du TOEC, BP 57611, 31076 Toulouse Cedex 03, France; 5 Service de Neurologie du CHRU de Nancy, 29, avenue du Maréchal de Lattre de Tassigny, Nancy, France; 6 Laval, France

**Keywords:** Epilepsy, Biomarkers, Mass spectrometry

## Abstract

Although epilepsy is considered a public health issue, the burden imposed by the unpredictability of seizures is mainly borne by the patients. Predicting seizures based on electroencephalography has had mixed success, and the idiosyncratic character of epilepsy makes a single method of detection or prediction for all patients almost impossible. To address this problem, we demonstrate herein that epileptic seizures can not only be detected by global chemometric analysis of data from selected ion flow tube mass spectrometry but also that a simple mathematical model makes it possible to predict these seizures (by up to 4 h 37 min in advance with 92% and 75% of samples correctly classified in training and leave-one-out-cross-validation, respectively). These findings should stimulate the development of non-invasive applications (e.g., electronic nose) for different types of epilepsy and thereby decrease of the unpredictability of epileptic seizures.

## Introduction

With an estimated 50 million people affected worldwide, epilepsy is recognized as a major public health concern^1^. It is characterized by repetitive, unpredictable seizures with the accompanying risk of injury and accident in addition to the associated psychosocial^2^ and comorbidity factors^3–5^. This disease affects the everyday life of patients; for example, by restricting their ability to drive any motorized vehicle, by hindering their capacity to obtain and retain meaningful employment, and by producing memory impairment, anxiety, depression, or even suicide. Approximately 30% of people with epilepsy are pharmaco-resistant. The manifest unpredictability of epileptic seizures places an immense psychological burden on patients and substantially compromises their quality of life^6,7^. Accurate prediction of seizures would not only reduce the burden on caregivers but also significantly improve the quality of life of patients, in particular by lessening their psychological stress. In addition, predicting seizures could prevent trauma or life-threatening accidents. Thus, accurate and reliable prediction of seizures has the potential to revolutionize the treatment of epilepsy. Furthermore, given that the clinical phenomenology^8^ of epileptic seizures varies widely, no generalized detection system exists to help people monitor their seizures^9^. Although electroencephalography is considered the gold standard for monitoring seizures, it remains difficult to use in everyday situations. Moreover, it generally offers a very short predictive time^10^ (on average 19 s before the onset of seizure^11^), allowing patients and caregivers insufficient time to react appropriately^12^.

A recent study showed that dogs could be trained to differentiate between the body odor emitted by patients during a seizure and the patients’ normal body odor, independently of the type or etiology of the seizure^13^. This discovery reveals that epileptic seizures have an olfactory signature, general to clinical phenomenological variations, which may open new prospects for managing epilepsy. Inspired by this canine study, we evaluate herein the feasibility of seizure detection based on the analysis of volatile organic compounds (VOCs) emitted by epileptic patients and investigate the kinetics thereof.

**Figure 1:**
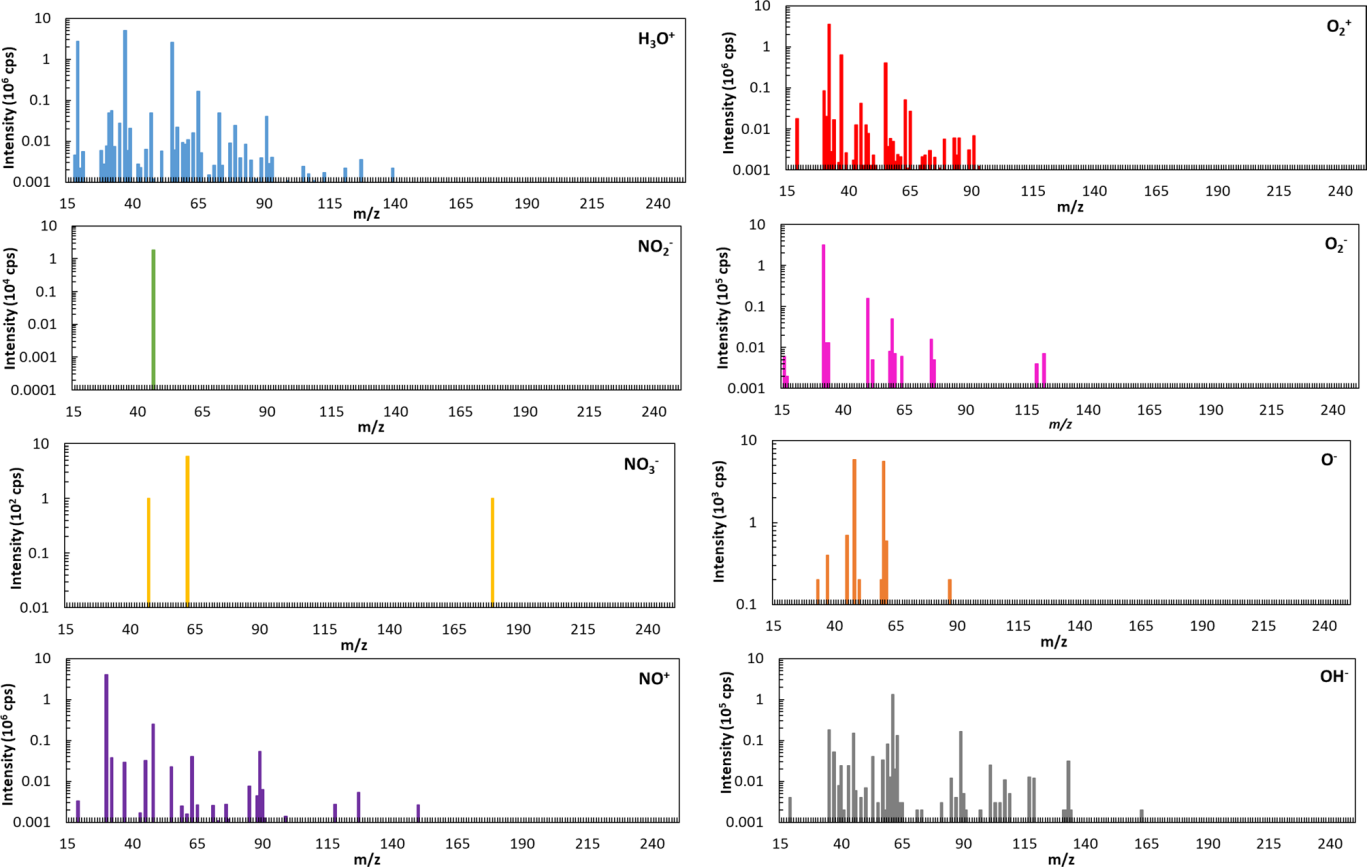
Example of SIFT-MS analysis of odor sample. These spectra reveal the ions generated by the ionization reaction of the sample with each of eight precursor ions (three positive ions: H_3_O^+^, NO^+^, O_2_^+^, and five negative ions: NO_2_^−^, NO_3_^−^, O_2_^−^, HO^−^, O^−^).

We use mass spectrometry to analyze the VOC profiles of epileptic patients. Selected ion flow tube mass spectrometry (SIFT-MS) is a gas-phase analytical method based on soft chemical ionization reactions coupled with direct mass spectrometry^14^. This analytical technique provides results in real-time, making it particularly appealing for use in a clinical setting. For some volatile compounds, the sensitivity of this method is better than parts per trillion^15^; it can also detect several target compounds simultaneously and can record global profiles (Fig. 1). We use a non-specific-recognition, learning-based model that involves relative comparisons between different modalities based on global VOC profiles.

This method has been applied previously in clinical studies to analyze exhaled breath and thereby distinguish between healthy patients and patients suffering from various conditions such as nonalcoholic fatty liver disease^16^, oesophagogastric cancer^17^, inflammatory bowel disease^18^, or pulmonary arterial hypertension^19^.

## Results

Odor-emitting samples were collected from 14 persons with a confirmed diagnosis of epilepsy, providing a mix of different body odors (see Supplementary Information). To collect the samples, the patients used a sterile cotton pad to wipe their hands, forehead, and the back of their neck. The cotton pad was then placed into a zip-locked bag and the patient was instructed to exhale into the bag before sealing it. These samples were collected in five situations: (a) a “seizure sample,” collected during or immediately after (< 5 min) an ictal event; (b) a “physical exercise sample,” collected immediately after (< 5 min) moderate exercise; (c) an “*n* − 1 sample,” which was the last sample collected before a seizure occurred; (d) an “*n* − 2 sample,” which was the sample obtained just before the *n* − 1 sample; and (e) an “inter-ictal sample,” collected at least six hours before or after the seizure. All samples were analyzed by SIFT-MS, and the results were processed by chemometric analysis.

### Classifying patients with epilepsy

A Welch’s modified *t*-test was applied to the first four principal components and to 61 averaged samples. The only test that approached significance was the one constructed for the third principal component. The SIFT-MS physical exercise and inter-ictal spectra collected from subjects differed sufficiently from the seizure, *n* − 1, and *n* − 2 spectra to justify classifying them separately (*n* = 61, *p* = 0.056). Based on these results, the status of each patient was categorized either as “seizure-related” (i.e., seizure, *n* − 1, or *n* − 2 samples) or “non-seizure-related” (i.e., inter-ictal or physical exercise samples).

### Seizure prediction based on classification tree analysis

The 1888 SIFT-MS variables served as input features for the models, and the two patient-status groups served as output. The model was based on a classification tree analysis of the 61 averaged samples. After optimization, the best model selected eight of the 1888 variables of the SIFT-MS spectra. This model correctly classified 92% and 75% of the samples based on the SIFT-MS spectra (see Table 1) in training and leave-one-out-cross-validation, respectively.

**Table 1:** Confusion matrix for the predicting condition of epileptic patients (classification-tree analysis) based on the SIFT-MS spectra. “Sensitivity” is defined as the percentage of seizure-related spectra that are well predicted by the model, whereas “specificity” is defined as the percentage of non-seizure-related spectra that are correctly rejected.

To	Inter-ictal and physical exercise	Seizure, n-1, n-2	Total	Well-classified samples (%)
From				
**Training**				
Inter-ictal and physical exercise	20	1	21	95.2 (Specificity)
Seizure, n-1, n-2	4	36	40	90 (Sensitivity)
Total	24	37	61	91.8
**Cross-validation**				
Inter-ictal and physical exercise	16	5	21	76.2 (Specificity)
Seizure, n-1, n-2	10	30	40	75 (Sensitivity)
Total	26	35	61	75.4

The SIFT-MS spectra of patients in an inter-ictal phase or during physical exercise were identified correctly by the algorithm in 20 of 21 cases (95.2%) and in 16 of 21 cases (76%) in leave-one-out cross validation. This rate is worth 90% (and 75% in cross-validation) for the “seizure-related” group.

The eight variables retained by the model are H_3_O^+^ 29 + ; H_3_O^+^ 38 + ; H_3_O^+^ 46 + ; H_3_O^+^ 61 + ; H_3_O^+^ 63 + ; H_3_O^+^ 137 + ; NO^+^ 79 + , and O_2_^+^ 77 + . Of the eight precursor ions tested by SIFT-MS, H_3_O^+^, NO^+^, and O_2_^+^ seemed to be relevant to discriminate between the seizure status of epileptic patients.

## Discussion

The results of this classification support the hypothesis that ictal samples differ from inter-ictal and physical exercise samples, which implies that a specific odor, or VOC profile, is associated with epileptic seizures, as suggested by the response of trained dogs to the odor of epileptic patients^13^. These results also indicate that the VOC profile does not depend on the type or etiology of epileptic seizures, reinforcing the hypothesis of an olfactory signature of epileptic seizures.

The rate of correct classification (92.5% of the samples in training and 75% in leave-one-out-cross-validation see Table 1).

Cross-validation has been implemented to ensure that our model does not suffer from overlearning, and that it will be able to make predictions on new data. The number of observations is low, which is why leave-one-out cross-validation is best suited. However, in a leave-one-out cross-validation, very similar training sets are formed, and very different test sets are formed. We will have almost the same model on each fold, and yet the quality of the predictions may vary a lot. Therefore it will be necessary to increase the number of observations to make the model more robust and to be able to validate it with an external data set or a k-folds cross validation.

This result is amongst the highest yet reported^10,11^ not only in terms of prediction delay but also in terms of sensitivity and specificity. However, these results are based on VOC profiles instead of on the more classical machine learning techniques applied to electroencephalography spectra. In addition, the results for detection of epileptic seizures are general to all types of seizures or epilepsy etiologies tested and not just to a specific type of seizure or to a precise cerebral location of the seizure onset. This is also one of the reasons why these performances are very encouraging.

The data for *n* − 2 samples are more similar to the data for *n* − 1 and seizure samples than to the data for inter-ictal and physical exercise samples, which suggests that this chemometric method may be used to predict seizures based on *n* − 2 samples. Given that the median time before seizure for *n* − 2 samples was 277 min, this result implies that seizures can be predicted over 4 h in advance with this method.

Although other methods^12,20^ have given good predictive results (> 90% for some), they are often too complex to process because they can involve significant data processing or *t*RNA expression analysis. In addition, they are usually invasive, requiring an implant, blood sampling, or the collection of other bodily fluids.

The limitations of this study include the small sample size, which was due to practical considerations. A larger sample would improve the learning capacity and validation of the model. In addition, because our sampling method was based on a whole-body approach, we could not determine the precise origin of the VOCs. Further research is thus required to determine the source of VOCs (e.g., skin, breath, sweat) or the possible involvement of sweat glands such as eccrine or apocrine glands. Additionally, we still do not know which biomarker underlies these findings because the pattern of VOCs that produces the VOC signature has yet to be defined.

In conclusion, although further research is needed to fully understand the mechanism of these findings, this first proof-of-concept study shows that epileptic seizures can be detected and predicted simply based on VOC analyses with a whole-spectrum approach. This method suggests that epileptic seizures can be predicted sufficiently in advance to be useful both for people with epilepsy and their caretakers.

Altogether, these results represent an opportunity to improve the life of people with epilepsy, independently of the patients’ demographics, type of seizure, or etiology. Furthermore, although this proof of concept is demonstrated herein by using SIFT-MS, these results pave the way for the development of monitoring technologies for everyday life. In fact, the increasing progress in wearable electronic noses should allow efficient, noninvasive sensors to be developed in a few years to finally guarantee the safety of epileptic patients.

## Materials and methods

### Ethics

This study was approved by the institutional review board of the OHS (Office of Social Hygiene) of Lorraine (France) for the collection techniques used to obtain odor samples from patients with epilepsy. The present research was noninvasive and did not involve pharmacological interventions. Thus, in accordance with the Ethics Committee’s guidelines, the adult patients, or guardians in the case of a minor, were only required to give informed written consent to allow their own or their child’s participation in the experiment prior to their inclusion in the study. Thus, Written Informed consent was obtained from participants and from guardians in the case of minors. The National Centre for Scientific Research (CNRS) Data Protection Official was consulted for this study and confirmed the validity of this approach. Information sanitization was fully anonymous.

### Odor samples

The patients recruited were from the medical and education institute (MEI) of the OHS Flavigny, Flavigny sur Moselle, France, which is a clinic for developmentally disabled children. Twenty patients were initially invited to participate after medical recommendation. The sampling occurred from March to May 2019. Six participants declined the invitation. Thus, odors were collected from 14 patients (7 women, 7 men) with ages varying from 10 to 43 years (mean: 27.7 ± 11.31 years old). All had a confirmed diagnosis of epilepsy (see Supplementary Information, Table 1) and none experienced psychogenic non-epileptic seizures. Psychogenic non-epileptic seizures, whether exclusive or in addition to epileptic seizures, were a factor of exclusion when recruiting patients on the basis of their medical diagnosis. Since all patients lived in the same MEI at the time of sampling, the diagnosis was further confirmed by the medical personnel. As in a previous study^13^, all patients received the same food without dietary restrictions. Since the seizures could occur at any time, the collection of epileptic seizure samples was random (night or day), whereas all other samples were collected during the day. To maintain a normal inter-individual variability corresponding to all types of habits reflective of everyday living conditions, patients were given no specific recommendation regarding hormonal contraception, perfume, smoking, etc.

### Odor-sampling procedure

The sampling procedure consisted in collecting several samples per day, with a three-hour interval between each sample. This method allowed us to collect five samples per day, from approximately 7 a.m. to 10 p.m., during inter-ictal or pre-ictal states. In addition, two types of samples were collected: (a) a “seizure sample,” collected during or immediately after (< 5 min) an ictal event and (b) a “physical exercise sample,” collected immediately after (< 5 min) moderate exercise. For this study, “moderate exercise” was defined as a 1 min interval during which the patient runs approximately 30 m and performs leg and arm movements such as step-touch and punches. Physical exercise samples served as control for movements or arousal, which can occur during a seizure^13,21,22^.

The five samples collected per day were divided into three types defined a posteriori: (c) the “*n* − 1 sample” is the last sample collected before a seizure occurs, (d) the “*n* − 2 sample” is the penultimate sample before a seizure occurs, and (e) the “inter-ictal sample” is collected at least 6 h before or after a seizure (to avoid pre- or post-ictal collection). For each type of sample, only a single sample was analyzed per patient. The average time between the *n* − 1 sample and a seizure was 206 min (standard deviation of 11.8 min). If the *n* − 1 and the seizure samples were acquired on two different days, they were considered extreme cases and discarded. The median time between the collection of *n* − 1 (*n* − 2) samples and the collection of seizure samples was 85 (277.5) minutes.

The sampling ended when a seizure occurred if a physical exercise sample had been collected before the seizure; otherwise, the physical exercise sample was collected the following day to avoid contaminating the sample by seizure odor. Thus, sampling lasted between one and five days, depending on the patient.

Odors were collected in accordance with the procedure used in previous studies:^13,23^ patients were instructed to use a sterile cotton pad (5 × 5 cm^2^, four-fold) to wipe their hands, forehead, and the back of their neck, allowing a multiplicity of odor origins. The cotton pad was then placed into a zip-locked bag (Ziplock brand, SC Johnson, Racine, WI, USA) and the patient was instructed to exhale into the bag before sealing it. Each bag was labeled with the patient’s code, the date, and time of collection. The samples were stored in a refrigerator at 4 °C until use (for an average of 60 days with a standard deviation of 9.7 days).

### Selected ion flow tube mass spectrometry

The method used in this study was based on that of Pasetto et al.^24^.

In the SIFT-MS device, the eight precursor ions were produced by microwave discharge. A single precursor ion was selected at a time by a first quadrupole mass spectrometer and then injected into a reaction chamber by flowing nitrogen (180 N mL min^−1^). The sample, heated to 37 °C for 10 h in a controlled-temperature oven, was introduced by a calibrated capillary (20 N mL min^−1^) into the reaction chamber, which was maintained at 115 °C and 0.07 kPa. The product ions generated from the ionization reaction and the precursor ions were quantified by a second quadrupole mass spectrometer^25^. In the full-mass mode, the second quadrupole mass spectrometer scanned over a large mass range (from 15 to 250 m/*z*) and calculated a count rate (signal intensity in counts per second) for each unit of *m*/*z*. This resulted in a full profile for each of the eight precursor ions, with 236 mass peaks summed for each precursor ion. Altogether, 1888 mass peaks were recorded.

Four full mass scans were recorded for each sample. None of the first scans were used for analysis because they served to purge the system between samples. The other three scans were averaged and used for further analyses.

### Statistical analyses

The approach proposed here was based on applying the chemometrics not of individual peaks, but of the entire spectra, as typically done with infrared spectroscopy^26^. This study thus focused on analyzing a global fingerprint.

First, we applied a principal component analysis (PCA), which is an orthogonal transformation, to convert the set of 1888 possibly correlated SIFT-MS variables into a set of linearly uncorrelated variables called principal components (PCs). PCA is defined so that the first PC accounts for the maximum possible variability in the SIFT-MS spectra, with the subsequent PCs accounting for less and less variability^27^. To develop the predictive model, we used a PCA to reduce the multidimensionality of the SIFT-MS spectral data^28^. The PCA was computed by using The Unscrambler (v. X; CAMO A/S, Oslo, Norway).

We selected the first PCs to account for 95% of the initial variability of the SIFT-MS spectra. The PCs were averaged for the triplicated analysis and then compared by using Welch's modified *t*-test^29^ regarding the patients’ state, as represented by the inter-ictal, physical exercise, seizure, n − 1 and *n* − 2 samples. The analysis was conducted by using Minitab (Minitab Inc, Statistical Software version 19, State College, PA, USA). The null hypothesis is that the average value of the dependent variable is the same for all patient states.

A C&RT classification tree analysis was then used to predict each patient’s state. The entropy measure served as a quality measure to split a node. This predictive model was developed from the 1888 variables of the SIFT-MS spectra by using Minitab version 19.2020.1. (Minitab Inc. PA, USA). The model was challenged with a leave-one-out-cross-validation. The quality of the model was evaluated by calculating classification errors and prediction accuracy from a confusion matrix^30,31^.

## Supplementary information

**Supplementary Information, Table 1.** Patient information at time of sampling.

## Data Availability

The data that support the findings of this study are available from the corresponding author upon reasonable request.
