# Supplementary material for: Prediction and detection of human epileptic seizures based on SIFT-MS chemometric data
Source: Sci Rep. 2020 Oct 27;10:18365. doi: 10.1038/s41598-020-75478-8 (PMC7591930; doi:10.1038/s41598-020-75478-8)
Supplement: Supplementary file 1 — Supplementary Information, Table 1. Patient information at time of sampling. [file 41598_2020_75478_MOESM1_ESM.docx]

Title: Prediction and detection of human epileptic seizures based on SIFT-MS chemometric data

**Authors:**

Amélie Catala^1,2^*, Cecile Levasseur-Garcia^3^, Marielle Pagès^4^, Jean-Luc Schaff^5^, Ugo Till^6^, Leticia Vitola Pasetto^3^, Martine Hausberger^2^, Hugo Cousillas^2^, Frederic Violleau^3‡^, Marine Grandgeorge^2‡^

**Supp. Info. Table 1** Person’s information at the time of the sampling

| ***Person*** | ***Sex*** | ***Age (year)*** | ***Etiology*** | ***Seizure type(s)*** | ***Seizure type sampled*** | ***Frequency of seizures (number per month)*** | ***Comorbidities*** |
| --- | --- | --- | --- | --- | --- | --- | --- |
| ***1*** | M | 13 | Lennox-Gastaut syndrome | myoclonic absence // tonic | n/a | 4 // 4 | cognitive impairment |
| ***2*** | M | 35 | Lennox-Gastaut syndrome | atypical absence // tonic clonic | tonic-clonic | 30 // 4 | behavioural disorders |
| ***4*** | F | 38 | neonatal anoxia | tonic-clonic | tonic-clonic | 4 | hiatal hernia and vision disorder |
| ***6*** | M | 18 | frontal dysplasia | motor and morpheic | n/a | 4 | urticaria and ADHD |
| ***7*** | M | 12 | frontal dysplasia | motor and morpheic | motor | 30 | behavioural disorders |
| ***9*** | F | 10 | Lennox-Gastaut syndrome | myoclonic absence // Tonic-clonic | myoclonic absence | 30 // 4 | no comorbidity |
| ***10*** | M | 25 | neonatal anoxia | tonic-clonic | tonic-clonic | 20 | gastrointestinal bleeding |
| ***11*** | M | 25 | anoxia post-SUDEP | myoclonic | myoclonic | 3 | cortical blindness and reflux esophagitis |
| ***12*** | M | 33 | neonatal anoxia | tonic-clonic | tonic-clonic | 10 | west syndrome and upper motor neuron syndrome |
| ***13*** | F | 43 | herpes simplex encephalitis | absence | absence | 5 | intellectual disability |
| ***17*** | F | 28 | neonatal anoxia | tonic-clonic | tonic-clonic | 7,5 | intellectual disability and pyramidal lobectomy |
| ***18*** | F | 26 | structural - West syndrome | tonic | tonic | 15 | light intellectual disability and clavicle and mandibular fracture |
| ***20*** | F | 41 | structural - encephalopathy | tonic-clonic | tonic-clonic | 7,5 | intellectual disability |
| ***21*** | F | 41 | Bourneville's tuberous sclerosis | tonic | tonic | 35 | no comorbidity |
